# Changes in Duration and Intensity of the World’s Top-Level Badminton Matches: A Consideration of the Increased Acute Injuries among Elite Women’s Singles Players

**DOI:** 10.3390/sports8020019

**Published:** 2020-02-08

**Authors:** Taro Iizuka, Kanako Hirano, Tomoaki Atomi, Miho Shimizu, Yoriko Atomi

**Affiliations:** 1 Material Health Science Laboratory, Graduate School of Engineering, Tokyo University of Agriculture and Technology, Tokyo 184-8588, Japan; t-iizuka@badminton.or.jp (T.I.); mshmz@cc.tuat.ac.jp (M.S.); 2 Nippon Badminton Association, Tokyo 160-0013, Japan; k-hirano@badminton.or.jp; 3 Department of Physical Therapy, Faculty of Health Sciences, Kyorin University, Tokyo 181-8612, Japan; tatomi@ks.kyorin-u.ac.jp

**Keywords:** badminton, performance analysis, timing variables, injury

## Abstract

The purpose of this study was to clarify whether there have been any specific changes in the characteristics of the world’s top-level women’s singles badminton matches compared to men’s singles matches after the current badminton scoring system was implemented in 2006. We compared the characteristics of the matches between the Super Series tournaments in 2007 and 2017. Match duration increased as the rally and rest times increased in both men’s and women’s singles matches. Specifically, in women’s singles, it was suggested that a further increase in physical demands because of the increased number of shots per second may have resulted in longer rest time in proportion to rally time. Moreover, increases in match duration (final eight, 53.3 ± 6.6 min; early rounds, 42.1 ± 3.6 min; P < 0.05) and number of shots per rally (final eight, 10.4 ± 1.2; early rounds, 8.7 ± 1.1; P < 0.05) in women’s singles were more prominent in the final eight rounds (quarterfinals, semifinals, and finals) than in the early rounds (rounds 1 and 2). The recent changes in characteristics of the world’s top-level badminton matches may account for the increased acute injuries that are frequently observed in elite women’s singles players. Thus, appropriate training programs are crucial to effectively improve performance and prevent injuries among elite badminton players.

## 1. Introduction

In 2006, the Badminton World Federation (BWF) changed the scoring system in badminton from the 15 points × 3 games (for women’s singles, 11 points × 3 games) service-point scoring system to the 21 points × 3 games rally-point scoring system. This scoring system was proposed to shorten the playing time and simplify the system for television viewers [1]. However, the match duration in recent top-level international tournaments increased under the current scoring system. A previous study showed that the duration in men’s singles matches in the 2012 Olympic Games in London significantly increased compared to that in the 2008 Olympic Games in Beijing [2]. Additionally, the authors indicated that rallies have become significantly longer and the number of shots per rally significantly increased in the 2012 London Olympic Games. Previous studies have demonstrated that badminton players face high physical demand in competitive matches [3,4,5]. Faude et al. (2007) reported that peak mean oxygen uptake (VO_2_) and heart rate (HR) in badminton singles matches were 73.3% and 89.0%, respectively [4]. With the aforementioned recent changes in the characteristics of the matches, the intensity of the world’s top-level men’s singles badminton matches may have also increased.

Badminton was one of the sports with the highest risk of injury during the 2012 London Olympic Games [6]. Acute injury during matches is frequently observed among elite female players [7], and this is especially evident in women’s singles. For example, anterior cruciate ligament (ACL) injuries were reported during the bronze medal match in the 2012 London Olympic Games and during the semi-final match in the 2016 Rio de Janeiro Olympic Games [8]. This suggests that changes in the world’s top-level women’s singles matches are remarkable, and the matches have possibly become more intense. We hypothesized that there have been specific changes in characteristics of the world’s top-level women’s singles matches compared to men’s singles matches. To plan appropriate training and injury prevention programs for elite badminton players, a clear understanding of the change in match characteristics in each discipline of the world’s top-level badminton matches is essential. Nevertheless, no research investigating the changes in the world’s top-level women’s singles matches has been conducted.

Therefore, in this study, we investigated the changes in characteristics of men’s singles and women’s singles matches in the world’s top-level badminton since the current scoring system was implemented in 2006. The analyses were two-fold. First, the duration of the matches in 12 BWF Super Series tournaments from 2007 to 2017 were compared and analysed. We considered that it was suitable to investigate the changes in characteristics of world’s top-level badminton matches by comparing the first edition (held in 2007, soon after the current scoring system was implemented in 2006) and the last edition (held in 2017) of the Super Series tournaments, which was widely regarded as the badminton competition with the highest standard worldwide [9]. Second, we investigated the changes in the timing variables of the matches that may have influenced the changes in match duration. We compared the timing variables of the matches between the Japan Open Super Series in 2007 and 2017, one of the 12 Super Series tournaments. We selected these tournaments for analysis since a sufficient number of men’s and women’s singles match videos were available to investigate the timing variables of the Super Series matches in 2007 and 2017.

## 2. Materials and Methods

### 2.1. Comparison of the Matches in Super Series 2007 and 2017

#### 2.1.1. Definition of Tournament Stage

In the Super Series tournaments, 32 players competed in the main draw of men’s singles and women’s singles events. The Super Series tournaments were composed of round 1, round 2, quarterfinals, semi-finals, and finals. Eight of the 32 players were seeded in each tournament according to their world ranking and the matches from the quarterfinals were often played between higher-ranked players; thus, matches tended to be longer and more intense from the quarterfinals. Intensity of the matches differs depending on the opposition [5,10]. Recent studies have indicated that match duration during the 2016 Rio Olympic Games was significantly different between the group stage and play-offs; the group stage served as the qualification round for the play-offs [11,12]. To conduct a thorough investigation of the changes in the characteristics of the world’s top-level badminton matches, we further classified the matches of the Super Series tournaments into two stages: early rounds—round 1 and round 2—and the final eight—quarterfinal, semi-final, and final.

#### 2.1.2. Match Duration

The official match statistics of the present and past tournaments sanctioned by the BWF, including Super Series tournaments, are available on the Tournament Software website [13,14]. We obtained match duration data for all the matches played in 12 Super Series tournaments in 2007 and 2017. Subsequently, we calculated the average match duration for the early rounds and for the final eight in each Super Series tournament. The number of matches included in the study is summarised in Table 1. 

### 2.2. Comparison of the Matches in the Japan Open 2007 and 2017

#### 2.2.1. Video Materials

Men’s singles and women’s singles matches in the Japan Open 2007 and 2017 were filmed and provided for this study by the Nippon Badminton Association. For the Japan Open 2007, 19 (early rounds) and six (final eight) match videos of men’s singles as well as 17 (early rounds) and seven (final eight) match videos of women’s singles were available. For the Japan Open 2017, 23 (early rounds) and seven (final eight) match videos of men’s singles and 23 (early rounds) and six (final eight) match videos of women’s singles were available.

#### 2.2.2. Match Analyses

The match videos were analysed using Sportscode Elite V10 (Hudl, Lincoln, NE, USA). The following timing variables were evaluated: rally time (time elapsed from the serve until the shuttlecock hits the ground), rest time (time elapsed from when the shuttlecock hits the ground until the next serve), work density (rally time divided by rest time), number of shots per rally (total number of times the shuttle is hit by both players during the rally time), number of shots per second (number of shots per rally divided by rally time), and effective playing time (EPT; rally time divided by rally + rest time multiplied by 100) [4,5,9,15]. In 2013, the instant review system for line judgement calls was implemented by the BWF. Under this system, each player has the right to request two revisions per game [12]. Since rest time including the instant review is usually >30 s, we excluded the rest time when a player requested revision for line calls as we aimed to achieve an identical comparison of the characteristics of the matches in 2007 and 2017.

### 2.3. Statistics

Data were presented as mean ± standard deviation. All statistical analyses were performed using SPSS version 19 (IBM, Armonk, NY, USA). The Shapiro-Wilk test was used to examine normal distributions for all variables. For the match duration and timing variables, a two-way analysis of variance (ANOVA) was conducted considering the year and tournament stage as factors. When appropriate, post hoc analyses were performed using the Bonferroni test. Partial eta squared (partial η^2^) served as the measure of the effect size in ANOVA. Differences in match duration between the early rounds and the final eight in 2007 and 2017 were compared using paired t-test for men’s singles and women’s singles matches. Statistical significance was set at P < 0.05.

## 3. Results

### 3.1. Comparison of the Matches in Super Series 2007 and 2017

**Figure 1:**
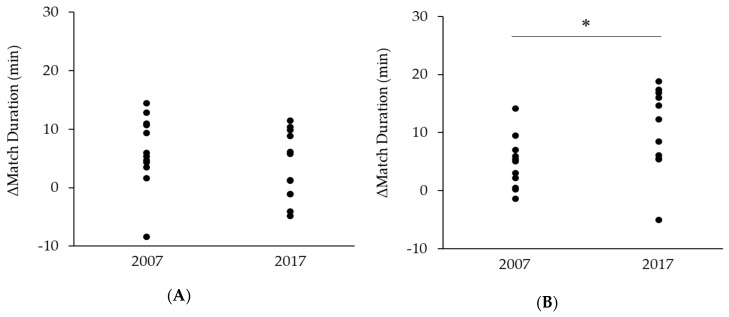
Comparison of the differences in match duration between the early rounds and the final eight in 2007 and 2017. (**A**) Men’s singles matches. (**B**) Women’s singles matches. * *P* <0.05.

Table 2 shows the difference in match duration in men’s singles of the Super Series distinguished by year and tournament stage. ANOVA revealed a significant main effect for year (P < 0.001). The result indicates that there was a significant increase in match duration from 2007 to 2017. However, no significant interaction between year and tournament stage was observed. Table 3 shows the difference in match duration in women’s singles of the Super Series distinguished by year and tournament stage. ANOVA revealed a significant interaction between year and tournament stage (P = 0.023), and post hoc comparisons revealed significant differences between 2007 and 2017 both in the early rounds (P = 0.035) and the final eight (P < 0.001). These results indicate that the increase in match duration from 2007 to 2017 was more prominent in the final eight than in the early rounds, despite the fact that a significant increase was observed in both tournament stages. In men’s singles, difference in match duration between the early rounds and the final eight was not significantly different between 2007 and 2017 (Figure 1A). In women’s singles, difference in match duration between the early rounds and the final eight had increased significantly from 2007 to 2017 (P = 0.008; Figure 1B).

### 3.2. Comparison of the Matches in Japan Open 2007 and 2017

Table 4 shows the differences in timing variables in men’s singles, distinguished by year and tournament stage. ANOVA for rally time (P = 0.047), rest time (P < 0.001), and number of shots per rally (P = 0.033) revealed a significant main effect for year. The results indicate that there was a significant increase from 2007 to 2017 for these variables. However, no significant interaction between year and tournament stage was observed. Table 5 shows the differences in timing variables in women’s singles distinguished by year and tournament stage. ANOVA for rally time (P = 0.001), rest time (P < 0.001), work density (P = 0.049), number of shots per second (P = 0.020), and EPT (P = 0.045) revealed a significant main effect for year. According to the results, rally time, rest time, and number of shots per second all showed significant increase from 2007 to 2017, while work density and EPT showed significant decrease from 2007 to 2017. No significant interaction between year and tournament stage was observed for these variables. ANOVA for number of shots per rally revealed a significant interaction between year and tournament stage (P = 0.018), and post hoc comparisons revealed a significant difference between 2007 and 2017 both in the early rounds (P = 0.036) and in the final eight (P < 0.001). In addition, a significant difference between the early rounds and the final eight in 2017 was observed (P = 0.003). These results indicate that the increase in number of shots per rally from 2007 to 2017 in the final eight were more prominent compared to that in the early rounds, despite the fact that a significant increase from 2007 to 2017 were observed in both tournament stages.

## 4. Discussion

The purpose of this study was to determine the changes in characteristics of the men’s singles and women’s singles matches in the world’s top-level badminton tournaments after the current badminton scoring system was implemented in 2006. We demonstrated that match duration of the Super Series tournaments increased both in men’s singles and women’s singles matches from 2007 to 2017. As Chiminazzo et al. (2018) indicated that the match duration in the 2016 Rio Olympic Games was different between group stage and play-offs [12], the match duration in men’s singles was significantly different between the early rounds and the final eight. However, the match duration increased by 10.9 min in the early rounds and 8.3 min in the final eight; thus, the match durations of the two tournament stages in 2017 were similar to those reported in previous studies that investigated the match duration of world’s top-level badminton matches under the current scoring system [12,15,16]. Gawin et al. (2015) reported that the average duration of men’s singles matches played between the world’s top 10 players was approximately 50 min [16]. In contrast, in women’s singles, although match duration significantly increased in both tournament stages, the extent of increase was quite different from each other. Differences in match duration in women’s singles between the early rounds and the final eight significantly increased from 4.9 min to 11.2 min in 2017, suggesting that the difference in physical demands between the two tournament stages has become greater in women’s singles.

For the changes in timing variables of the matches based on the Japan Open 2007 to 2017, rally time and rest time increased significantly regardless of tournament stage in both men’s and women’s singles. Cabello Manrique et al. (2003) found that longer rest times are required to recover from longer rallies [5]. However, when we examined the balance between the length of rally time and rest time, significant decreases in work density and EPT were observed only in women’s singles. This may be due to the increase in number of shots per second that was observed in women’s singles. A previous study reported that flat or downward trajectory shots with faster and more explosive characteristics are more frequently used in men’s singles than in women’s singles, which could be accounted for by the difference in physical ability of the players to produce a large striking force [17]. Nevertheless, the increase in the number of shots per second in women’s singles matches suggests that the physical ability of women’s singles players has increased and that they could produce faster shots with flat and downward trajectories and take points more aggressively. These increases in the frequency and duration of rallies resulted in more intense women’s singles matches and thus, required longer rest time in proportion to the length of rallies. However, Laffaye et al. (2015) reported that metabolic constraints may not be the only factor in determining the length of rest time [15]. As the matches become more competitive, players would also need more time to strategize to gain a point in the following rally.

Compared to the early rounds, the final eight in women’s singles matches had a marked increase in number of shots per rally resulting from the combination of increase in rally time and in number of shots per second. Abian et al. (2014) suggested that longer rallies and shortened stroke time combined with increased number of shots per rally make badminton matches more competitive [2]. During such matches, players are highly loaded and could get more tired as they are required to continue making quick starts, stops, and direction changes [18]. Valldecabres et al. (2018) showed that movement control and strategy during lunges are influenced by fatigue and suggested that these changes could be risk factors for injury among badminton players [19]. Therefore, especially in women’s singles matches played between higher-ranked players, more attention should be paid in the preparation of the players to prevent injury. Benjaminse et al. (2019) suggested that training to resist fatigue could be effective for injury prevention as fatigue may play a crucial role in sustaining an ACL injury [20]. 

Since 2018, the Super Series has been replaced by the BWF World Tour. In the World Tour, the number of high-grade tournaments corresponding to the Super Series expanded from 12 to 15 and the world’s top-level players are encouraged to participate in more tournaments each year. Indeed, in the new ranking system, men’s and women’s singles players in the top 15 of the world ranking at the end of a year are required to play in at least 12 of the 15 high-grade World Tour tournaments (the BWF World Tour Super 500 and above) in the following year. Therefore, given that the world’s top-level badminton matches have become more physically demanding, planning strategies to effectively recover in between the matches and tournaments would be necessary for the players to avoid accumulating fatigue, which can be a risk factor for injury.

This study has several limitations. First, we investigated the changes in timing variables of the matches by comparing Japan Open 2007 and 2017 instead of comparing all the matches from the 12 Super Series tournaments in 2007 and 2017. Therefore, our results regarding changes in timing variables of the matches may not fully explain the increase in match duration in the Super Series tournaments. Second, although our novel finding that the number of shots per second increased in recent women’s singles matches would be significant in planning appropriate training and injury prevention programs, the reason for the increase was not elaborated as we did not conduct a notational analysis of the matches. Laffaye et al. (2015) suggested that high shot frequency is one of the characteristics of recent badminton matches, and this parameter should be considered for a more practical training given the recent highly competitive matches [15]. Third, because of the paucity of literature, we could not describe how the injuries among the world’s top-level men’s and women’s singles badminton players increased from 2007 to 2017. It is necessary to further investigate the variation of acute injury incidence in elite badminton players. 

In conclusion, based on the results of this study, it can be stated that match duration has significantly increased in men’s and women’s singles matches, which could be associated with the increase in rally time and rest time. It was suggested that women’s singles matches have become more intense as the number of shots per second increased in addition to the increase in rally time, which in turn possibly resulted in a further increase in rest time in proportion to rally time. Furthermore, compared to the early rounds, the final eight had increased match durations and number of shots per rally in women’s singles matches. From these findings, not only length of rallies but also shot frequency and number of shots per rally should be taken into account to plan effective training to improve the performance of elite women’s singles players. Previous studies have suggested that training should be different between singles and doubles disciplines as the physical demands vary [10,21,22]. However, our results suggest that each discipline may require changes in the training, given the course of changes in the characteristics of the matches.

## Figures and Tables

**Table:** (**A**)

			Number of Matches			
			MS		WS	
Tournament	Place	Period	Early Rounds	Final Eight	Early Rounds	Final Eight
**Malaysia Open**	Kuala Lumpur	2007.1.16–1.21	24	6	24	7
**Korea Open**	Seoul	2007.1.23–1.28	23	7	24	7
**All England Open**	Birmingham	2007.3.6–3.11	24	7	24	6
**Swiss Open**	Basel	2007.3.12–3.18	24	6	24	7
**Singapore Open**	Singapore	2007.5.1–5.6	24	7	24	7
**Indonesia Open**	Jakarta	2007.5.7–5.13	24	7	23	7
**China Masters**	Chengdu	2007.7.10–7.15	24	7	23	7
**Japan Open**	Tokyo	2007.9.11–9.16	24	7	24	7
**Denmark Open**	Odense	2007.10.23–10.28	24	7	24	7
**French Open**	Paris	2007.10.30–11.4	24	7	24	7
**China Open**	Guangzhou	2007.11.20–11.25	24	6	24	7
**Hong Kong Open**	Hong Kong	2007.11.26–12.2	23	6	23	7
**Total**			286	80	285	83

**Table:** (**B**)

			Number of Matches			
			MS		WS	
Tournament	Place	Period	Early Rounds	Final Eight	Early Rounds	Final Eight
**All England Open**	Birmingham	2017.3.7–3.12	23	7	24	7
**India Open**	New Delhi	2017.3.28–4.2	23	7	22	7
**Malaysia Open**	Kuala Lumpur	2017.4.4–4.9	24	6	24	7
**Singapore Open**	Singapore	2017.4.11–4.16	24	7	24	7
**Indonesia Open**	Jakarta	2017.6.12–6.18	24	7	23	7
**Australian Open**	Sydney	2017.6.20–6.25	23	7	24	7
**Korea Open**	Seoul	2017.9.12–9.17	24	7	24	7
**Japan Open**	Tokyo	2017.9.19–9.24	24	7	23	6
**Denmark Open**	Odense	2017.10.17–10.22	24	7	22	7
**French Open**	Paris	2017.10.24–10.29	23	6	24	7
**China Open**	Fuzhou	2017.11.14–11.19	24	7	23	7
**Hong Kong Open**	Hong Kong	2017.11.21–11.26	23	7	23	7
**Total**			283	82	280	83

Matches that were not completed were not included. MS: Men’s Singles; WS: Women’s Singles. Early rounds: round 1 and round 2; Final eight: quarterfinal, semi-final, and final

**Table 2:** Match duration in the early rounds and in the final eight of men’s singles badminton matches in the Super Series (2007 and 2017).

	Early Rounds		Final Eight		Main Effect		Interaction
	2007	2017	2007	2017	Year	Stage	
Match duration (min)	38.2	49.1	44.5	52.8	P < 0.001 (partial η^2^ = 0.576)	P < 0.001 (partial η^2^ = 0.273)	P = 0.290 (partial η^2^ = 0.025)
	(2.8)	(3.7)	(5.1)	(5.0)			

Data are presented as mean (standard deviation). Early rounds: round 1 and round 2; Final eight: quarterfinal, semi-final, and final.

**Table 3:** Match duration in the early rounds and in the final eight of women’s singles badminton matches in the Super Series (2007 and 2017).

	Early Rounds		Final Eight		Main Effect		Interaction
	2007	2017	2007	2017	Year	Stage	
Match duration (min)	38.0	42.1 *	42.9 ^#^	53.3 *^,#^	P < 0.001 (partial η^2^ = 0.401)	P < 0.001 (partial η^2^ = 0.448)	P = 0.023 (partial η^2^ = 0.112)
	(3.3)	(3.6)	(4.2)	(6.6)			

Data are presented as mean (standard deviation). Early rounds: round 1 and round 2; Final eight: quarterfinal, semifinal, and final. * P < 0.05 vs. 2007 compared within the same tournament stage. # P < 0.05 vs. early rounds compared within the same year.

**Table 4:** Timing variables in the early rounds and in the final eight of men’s singles matches in the Super Series (2007 and 2017).

	Early Rounds		Final Eight		Main Effect		Interaction
	2007	2017	2007	2017	Year	Stage	
Rally time (s)	8.4	10.0	9.3	9.8	P = 0.047 (partial η^2^ = 0.075)	P = 0.538 (partial η^2^ = 0.007)	P = 0.326 (partial η^2^ = 0.019)
	(1.6)	(1.9)	(1.1)	(1.6)			
Rest time (s)	17.8	21.9	20.5	22.9	P < 0.001 (partial η^2^ = 0.220)	P = 0.038 (partial η^2^ = 0.081)	P = 0.348 (partial η^2^ = 0.017)
	(2.8)	(3.0)	(2.0)	(1.9)			
Work density	0.47	0.46	0.46	0.43	P = 0.323 (partial η^2^ = 0.019)	P = 0.347 (partial η^2^ = 0.016)	P = 0.703 (partial η^2^ = 0.003)
	(0.07)	(0.06)	(0.11)	(0.07)			
Number of shots per rally	9.0	10.8	10.0	10.5	P = 0.033 (partial η^2^ = 0.086)	P = 0.527 (partial η^2^ = 0.008)	P = 0.192 (partial η^2^ = 0.033)
	(1.5)	(1.8)	(0.8)	(1.6)			
Number of shots per second	1.08	1.09	1.09	1.07	P = 0.576 (partial η^2^ = 0.006)	P = 0.731 (partial η^2^ = 0.002)	P = 0.366 (partial η^2^ = 0.016)
	(0.04)	(0.04)	(0.04)	(0.05)			
EPT	31.9	31.3	31.3	29.9	P = 0.341 (partial η^2^ = 0.018)	P = 0.318 (partial η^2^ = 0.020)	P = 0.737 (partial η^2^ = 0.002)
	(3.0)	(4.0)	(4.6)	(3.2)			

Data are presented as mean (standard deviation). EPT: effective playing time. Early rounds: round 1 and round 2; Final eight: quarterfinal, semifinal, and final.

**Table 5:** Timing variables in the early rounds and in the final eight of women’s singles matches in the Super Series (2007 and 2017).

	Early Rounds		Final Eight		Main Effect		Interaction
	2007	2017	2007	2017	Year	Stage	
Rally time (s)	8.4	9.0	8.0	10.0	P = 0.001 (partial η^2^ = 0.210)	P = 0.338 (partial η^2^ = 0.019)	P = 0.060 (partial η^2^ = 0.070)
	(1.0)	(1.1)	(1.4)	(1.5)			
Rest time (s)	17.7	21.5	19.2	25.2	P < 0.001 (partial η^2^ = 0.422)	P = 0.003 (partial η^2^ = 0.169)	P = 0.179 (partial η^2^ = 0.037)
	(2.8)	(2.3)	(2.6)	(3.1)			
Work density	0.48	0.42	0.42	0.40	P = 0.049 (partial η^2^ = 0.077)	P = 0.033 (partial η^2^ = 0.089)	P = 0.210 (partial η^2^ = 0.032)
	(0.05)	(0.05)	(0.04)	(0.08)			
Number of shots per rally	8.0	8.7 *	7.8	10.4 *^,#^	P < 0.001 (partial η^2^ = 0.301)	P = 0.045 (partial η^2^ = 0.079)	P = 0.018 (partial η^2^ = 0.109)
	(1.0)	(1.1)	(1.4)	(1.2)			
Number of shots per second	0.96	0.98	0.98	1.04	P = 0.020 (partial η^2^ = 0.105)	P = 0.012 (partial η^2^ = 0.122)	P = 0.278 (partial η^2^ = 0.024)
	(0.05)	(0.05)	(0.03)	(0.06)			
EPT	32.3	29.5	29.4	28.6	P = 0.045 (partial η^2^ = 0.079)	P = 0.035 (partial η^2^ = 0.088)	P = 0.268 (partial η^2^ = 0.025)
	(2.5)	(2.7)	(2.0)	(4.2)			

Data are presented as mean (standard deviation). EPT: effective playing time. Early rounds: round 1 and round 2; Final eight: quarterfinal, semifinal, and final. * P < 0.05 vs. 2007 compared within the same tournament stage. # P < 0.05 vs. Early Rounds compared within the same year.
